# Fly piRNA biogenesis: tap dancing with Tej

**DOI:** 10.1186/s12915-014-0077-1

**Published:** 2014-10-06

**Authors:** Zhaolin Yang, Ramesh S Pillai

**Affiliations:** European Molecular Biology Laboratory, Grenoble Outstation, University Grenoble Alpes-EMBL-CNRS, 71 avenue des Martyrs, Grenoble, 38042 France; Unit for Virus Host-Cell Interactions, University Grenoble Alpes-EMBL-CNRS, 71 avenue des Martyrs, Grenoble, 38042 France

## Abstract

Piwi-interacting RNAs (piRNAs) protect animal germlines from the deleterious effects of transposon activity. Unlike other small RNA classes like microRNAs (miRNAs) and small interfering RNAs (siRNAs), an exceptionally large number of factors are implicated in the biogenesis of piRNAs. Kai *et al.* have now added another one to this growing list, which we discuss in the overall context of our current knowledge of the piRNA biogenesis pathway in the *Drosophila* ovarian germline.

See research article: http://www.biomedcentral.com/1741-7007/12/61.

In recent years, the Piwi-interacting RNA (piRNA) pathway has come to be recognized as the guardian of the germline genome, as it silences mobile genetic elements in animal gonads. Transposon mobility is potentially detrimental to genome integrity as it can lead to mutations, causing a block in germ cell development. Consistently, piRNA pathway mutant animals activate transposons in the gonads and are invariably sterile. A great deal of research - much of it in the *Drosophila* ovarian environment (Figure 1a) - has gone into understanding how piRNAs are made and how they function, but major questions still remain. In this issue, Kai and colleagues report a new factor that is involved in the *Drosophila* piRNA biogenesis pathway [1].

**Figure 1:**
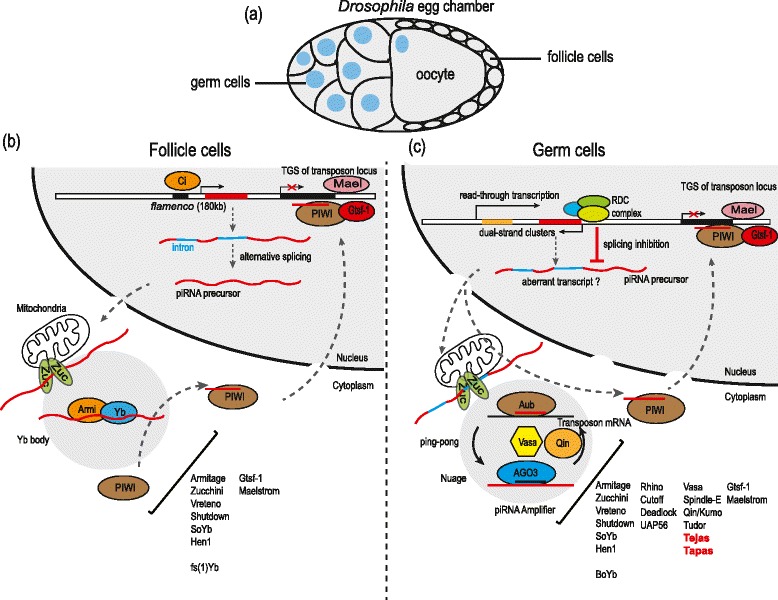
**piRNA pathways in the** ***Drosophila*** **ovary. (a)** A schematic representation of a fly egg chamber showing the germline (the single developing oocyte and 15 nurse cells) surrounded by a single layer of somatic follicle cells. **(b)** The unistrand piRNA cluster (piRNAs arising from only one strand) *flamenco* in the somatic follicle cells is transcribed by the transcription factor Ci. This approximately 180 kb long precursor is alternatively spliced and exported to the cytoplasm for processing in the nuage or Yb bodies. Nuage are perinuclear granules that contain most known piRNA biogenesis factors and are usually in the vicinity of mitochondria. Conversion of the long precursor RNA into approximately 24 nucleotide piRNAs takes place by an unknown mechanism called primary processing. Only PIWI that is loaded with piRNAs becomes licensed for import into the nucleus. Nuclear PIWI suppresses transposons via transcriptional gene silencing (TGS). A few nuclear factors (Gtsf-1 and Maelstrom) are known to be essential for TGS, but not for piRNA biogenesis. All known factors involved in the somatic pathway are listed. **(c)** The germline has both primary and secondary piRNA biogenesis pathways. Transcription of the dual-strand cluster (piRNAs arising from both strands) is thought to be non-canonical and arises from RNA polymerase II read-through from neighboring transcription units. A nuclear complex composed of Rhino-Deadlock-Cutoff (RDC) is essential for their transcription and is implicated in suppressing splicing and other RNA processing events [2]. Such ′aberrant′ transcripts are swept into the piRNA processing pathway. These are then processed in the cytoplasm to load all the three fly Piwi proteins. While many factors are shared with the follicle cells, a number of factors are unique to the germline compartment and they are required for the ‘ping-pong’ cycle, also known as secondary piRNA biogenesis. The RNA helicase Vasa orchestrates assembly of a piRNA Amplifier complex that generates new secondary piRNAs. Cytoplasmic Piwi proteins Aub and Ago3 destroy transposons by endonucleolytic cleavage, while PIWI acts in the nuclear compartment via TGS.

## Piwi proteins and Piwi-interacting RNAs

As with other small RNAs, piRNAs associate with Argonaute proteins, albeit a different class called Piwi (Box 1); however, early on it was realized that piRNA biogenesis is very different from that of other small RNAs. The most obvious distinguishable difference was the longer size (24 to 30 nucleotides) of piRNAs, which was inconsistent with a role for the Dicer endonuclease. Dicer acts as a molecular ruler to process double-stranded RNA precursors into approximately 21 nucleotide small RNAs, so structurally it would be impossible to produce longer small RNA species. Indeed, by the use of genetic mutants, Dicer was shown to be dispensable for piRNA biogenesis in fruitflies and fish.

Another difference is the sheer complexity of piRNA sequences. Unlike the several hundred miRNA sequences that were initially catalogued through conventional cloning, high-throughput ′deep′ sequencing methodologies had to be employed to reveal the full spectrum of piRNA sequences. In fact, millions of individual piRNAs can be identified and their mapping to the genome led to the identification of a few hundred hotspots where they cluster together. These so-called piRNA clusters usually range from 50 to 100 kilobases (kb) and are the sources of most piRNAs [3].

## Biogenesis pathways

Based on piRNA sequence signatures and biogenesis factor requirements, two distinct cytoplasmic piRNA biogenesis pathways have been recognized: primary and secondary biogenesis. In the *Drosophila* ovarian environment, primary biogenesis is operational in both the somatic follicle cells and the germline, while secondary biogenesis - known as the ‘ping-pong’ cycle - is specific to the germline.

### Primary piRNA biogenesis

A combination of transcriptome sequencing, mapping of transcription factor binding sites, and genetic analyses have revealed that piRNA clusters are transcribed into long single-stranded transcripts [4]. Such precursors are then exported to cytoplasmic granules called nuage (also called Yb bodies in the fruitfly ovarian soma) where most piRNA biogenesis factors reside. Primary processing describes the path taken by such precursors to end up as approximately 24 nucleotide primary piRNAs [3]. In the ovarian soma, a single type of Piwi protein, PIWI, receives primary piRNAs. Although we do not have a clear picture, it is believed that the precursor RNA is randomly broken up by unknown nucleases into fragments, which are then loaded onto a Piwi protein, such that the 5′ end of the fragment (pre-piRNA) is inserted into its MID domain (Box 1). Primary piRNAs display a strong preference for a 5′ uridine (1U-bias), but primary processing generates piRNAs without any such preference. So it is likely that 1U-bias is an outcome of the nucleotide preference of the MID domain that allows the Piwi protein to bind and enrich certain piRNAs. Subsequently, another unknown 3′-5′ exonuclease (tentatively called Trimmer) cleaves the 3′ end to generate the mature piRNA. The 3′ end is then modified by a 2′-*O*-methyl modification catalyzed by the RNA methyltransferase Hen1.

To date, Zucchini is the only protein known to act as a nuclease during primary processing. Zucchini homodimerizes, presumably when anchored on the outer membrane of mitochondria, to form an endonuclease active on single-stranded RNAs [5], but its exact substrate or product in the piRNA pathway is not known (Figure 1b). Other primary piRNA biogenesis factors include RNA helicases Armitage (Armi) and Yb, the Hsp90 co-chaperone Shutdown, and an RNA-binding protein Vreteno. Molecular details of how these factors function are yet to be determined.

One major question to consider is how the primary processing machinery distinguishes Pol II-transcribed piRNA cluster transcripts from other capped and polyadenylated noncoding RNAs. One possibility is that the nuclear history of transcription from the cluster promoter is conveyed by a co-transcriptionally loaded RNA-binding protein. However, the transcription factor Cubitus interruptus (Ci) that transcribes the *flamenco* cluster in the ovarian soma may not be enough to signal this, as Ci transcribes other protein-coding genes that do not enter the piRNA pathway. Alternatively, the information might be contained in the precursor RNA itself in the form of a specific sequence or an RNA structural element. The latter possibility is attractive given that the piRNA processing machinery is largely conserved across the animal kingdom, so it might identify conserved features in piRNA precursors.

It is interesting to note that before reaching the nuage, cluster transcripts like that of *flamenco* may transit through another perinuclear structure called Dot-COM/Flam-body that is in the proximity of Yb bodies [6]. Once PIWI is loaded, it becomes licensed for nuclear import. The bound piRNAs are then proposed to identify target genomic loci by annealing to nascent transcripts, eventually leading to deposition of silent H3K9me3 marks on the target chromatin. To enable this transcriptional gene silencing (TGS), PIWI collaborates with a zinc-finger protein Gtsf-1 and a HMG box-containing protein Maelstrom.

### Secondary piRNA biogenesis: the ‘ping-pong’ cycle

Germline piRNA biogenesis is more complex than in somatic cells as all three Piwi proteins (Aubergine, Ago3 and PIWI) are involved, as opposed to PIWI alone. Most factors implicated in the follicle cells for primary processing also act in the germline, loading primary 1U-containing piRNAs into Aubergine (Aub) and PIWI. This results in the loading of essentially antisense repeat piRNAs into the two proteins [3]. Thus, the primary processing machinery is somehow able to distinguish the different Piwi proteins present in the germline and deliver piRNAs only into specific proteins. Once loaded, PIWI goes into the nucleus, while Aub remains in the cytoplasm to recognize and slice complementary cluster transcripts or sense transposon targets. The latter step leads to post-transcriptional gene silencing (PTGS) of transposable elements in the cytoplasm. In a process unique to the germline, a specialized machinery of proteins then converts one of the cleavage fragments into a sense-oriented secondary piRNA that is loaded into Ago3 [3]. Such sense Ago3 piRNAs can then target antisense cluster transcripts to slice and generate the exactly same antisense Aub-bound piRNAs that initiated the process. This feed-forward piRNA amplification loop - the ping-pong cycle - functions as an adaptive arm of the transposon defense system, as every time a target is destroyed more (silencing) antisense piRNAs are generated.

Secondary piRNA biogenesis links post-transcriptional gene silencing of transposon transcripts to biogenesis of a new piRNA, and this requires a number of factors unique to the germline environment (Figure 1c). Recent studies in *Bombyx mori* (Silkworm) BmN4 cell culture have shed light on the molecular role of the conserved DEAD box RNA helicase Vasa in linking the two events [7]. Vasa is shown to function as an ATP-regulated RNA clamp that anchors a piRNA Amplifier complex on transposon transcripts. Such a complex is composed of Vasa, the two ping-pong Piwi partners, the Tudor domain protein Qin/Kumo, antisense piRNAs and complementary sense transposon transcripts. The purpose of the Amplifier complex is to bring the two ping-pong partners into close proximity such that an Aub-generated transposon cleavage fragment can be transferred to Ago3. Such an exchange is triggered by hydrolysis of ATP within Vasa. The multiple Tudor domain protein Qin/Kumo is known to promote Aub-Ago3 interactions to ensure heterotypic ping-pong between the two proteins [8]. The specificity of the process is evident from the fact that Ago3 fails to become loaded with piRNAs in cell culture environments where only primary processing is active. In fact, Krimper is shown to sequester unloaded Ago3 in specialized Krimp bodies, presumably to prevent illegitimate entry of RNA sequences into Ago3. A number of other factors are shown to be essential for the ping-pong cycle, but their molecular functions are not clearly understood (Figure 1c).

## Tudor proteins

Krimper belongs to one of the largest identifiable group of piRNA biogenesis factors: the Tudor domain-containing proteins. They are defined by the presence of a Tudor domain, which contains an aromatic cage that recognizes methylated arginine or lysine residues. Piwi proteins and Vasa are shown to be symmetrically dimethylated at arginines, and these are recognized by Tudor domains [9]. Several Tudor proteins have multiple Tudor domains that can associate with different partners at the same time, suggesting a scaffolding function. Many of them have additional domains that provide functionalities like RNA helicase activity, RNA binding and E3 ligase activity. In this issue, a study from the Toshie Kai lab reveals the involvement of a new Tudor domain protein, Tapas (Tap), in the piRNA biogenesis.

Tap is a multiple domain protein composed of a Lotus domain and three Tudor domains. It is a paralog of Tejas (Tej), which is known to be essential for secondary piRNA biogenesis and fertility in the fly ovarian germline [10]. Like Tej, Tap is also exclusively detected in the germline and accumulates in the nuage. While flies lacking *tap* are fertile, they display mild activation of retrotransposons that are normally under control of germline piRNAs. In fact, germline piRNA levels and sequence signatures consistent with an active ping-pong cycle are slightly reduced in the mutant. The authors demonstrate biochemical interactions between the protein and other secondary piRNA biogenesis factors like Vasa, Spn-E, Aub and Ago3, indicating that Tap likely has a scaffolding role during assembly of complexes required for the ping-pong cycle.

Interestingly, the authors reveal a biochemical interaction between Tap and Tej. Given this, and the fact that *tap* mutant has a mild phenotype, the authors examined the *tej-tap* double mutant flies, which turned out to be sterile. Indeed, the double mutants display stronger activation of transposons, changes in piRNA levels, and mislocalization of nuage components than any of the single mutants. Despite this apparent cooperative effect, the two proteins have distinct roles as they cannot functionally complement each other. So Tej and Tap are proposed to function synergistically in the ping-pong cycle. One strong display of this synergistic function is their role in nuclear accumulation of PIWI in the germline. While individual *tej* and *tap* mutants are able to support nuclear localization of PIWI, the protein remained in the cytoplasm in the double mutant, presumably due to lack of loaded piRNAs. This is likely a consequence of the strong disruption in localization of various nuage factors in the double mutant. This partnership between Tej and Tap is conserved, as mouse mutants lacking their orthologues Tdrd5 and Tdrd7 display infertility, with mice lacking Tdrd5 derepressing retrotransposons.

Research so far has yielded a rich harvest of factors implicated in piRNA biogenesis, but key nucleases are still missing in the pathway. The use of available cell culture models should allow mechanistic study of these factors without the confounding effects of arrested germline development seen in mutant animals. Furthermore, application of a combination of biochemical, structural and genetic analyses of targeted point mutants should shed light on their molecular roles in the pathway.

### Box 1. Piwi and the Argonautes

Small RNAs are bound by proteins belonging to the Argonaute family, which can be broadly classified into the ubiquitously expressed AGO clade that binds approximately 21 nucleotide microRNAs (miRNAs) and small interfering RNAs (siRNAs), and the animal-specific Piwi-clade, which is mainly restricted to the gonads. This mirrors the distribution of their respective associated small RNAs: miRNAs and siRNAs are detected in almost all organisms and tissues, whereas piRNAs are specific to animals and found in the gonads. Argonaute family proteins are built to bind small RNAs; they contain characteristic PAZ, MID and PIWI modules. The 5′ and 3′ ends of the bound RNA are contacted by the MID and PAZ modules, respectively, such that the RNA bases are available for base pairing with complementary sequences. Perfect match with a target leads to its endonucleolytic cleavage by the PIWI module. Thus, some Argonautes are small RNA-guided nucleases (slicers) that can post-transcriptionally control target RNA levels. Nuclear Argonautes function by recruiting complexes that promote either histone or DNA methylation at target genomic loci.
